# Iron Loading and Overloading due to Ineffective Erythropoiesis

**DOI:** 10.1155/2010/358283

**Published:** 2010-05-11

**Authors:** Toshihiko Tanno, Jeffery L. Miller

**Affiliations:** Molecular Medicine Branch, NIDDK, National Institutes of Health, Bethesda, MD 20892, USA

## Abstract

Erythropoiesis describes the hematopoietic process of cell proliferation and differentiation that results in the production of mature circulating erythrocytes. Adult humans produce 200 billion erythrocytes daily, and approximately 1 billion iron molecules are incorporated into the hemoglobin contained within each erythrocyte. Thus, iron usage for the hemoglobin production is a primary regulator of plasma iron supply and demand. In many anemias, additional sources of iron from diet and tissue stores are needed to meet the erythroid demand. Among a subset of anemias that arise from ineffective erythropoiesis, iron absorption and accumulation in the tissues increases to levels that are in excess of erythropoiesis demand even in the absence of transfusion. The mechanisms responsible for iron overloading due to ineffective erythropoiesis are not fully understood. Based upon data that is currently available, it is proposed in this review that loading and overloading of iron can be regulated by distinct or combined mechanisms associated with erythropoiesis. The concept of erythroid regulation of iron is broadened to include both physiological and pathological hepcidin suppression in cases of ineffective erythropoiesis.

## 1. Introduction

In the absence of blood transfusions, hereditary mutations play the primary role in most syndromes of iron overload [1]. The genetic bases for several inherited forms of hemochromatosis were identified and experimentally confirmed as genes involved in iron regulation. These discoveries subsequently led to major advances in understanding iron biology [2]. Distinct from iron-regulating genes and their products, a group of erythroid disorders that demonstrate ineffective erythropoiesis also manifest a hemochromatosis phenotype. While tissue iron overload is a shared feature of these erythroid disorders, there is no evidence for a shared genetic mutation. Instead, the ineffective erythropoiesis itself seems to cause iron accumulation and eventual overload. Iron loading in the liver and other tissues proceeds well beyond the levels needed to support erythropoiesis. Based upon mapping of the human genome and the discovery of hepcidin, new mechanisms are being explored for physiological and pathological regulation of iron associated with ineffective erythropoiesis. 

## 2. Understanding Ineffective Erythropoiesis

Erythrocytes serve a major function of oxygen transport and delivery throughout the body. Erythrocyte production is appropriately driven by inadequate delivery of oxygen to the tissues. The reduced tissue oxygen levels are sensed by peritubular cells in the renal cortex and outer medulla [3]. In response, those kidney cells express and secrete erythropoietin (EPO) [4]. Plasma EPO is transported to the bone marrow in order to promote the production of new erythrocytes. The new erythrocytes are produced through the process whereby erythroblasts respond to erythropoietin via proliferation and differentiation over the course of several days. During erythropoiesis, large amounts of iron are needed to produce hemoglobin. Importantly, transferrin-bound iron is endocytosed into erythroblasts after binding to the plasma membrane receptors [5–8]. High levels of membrane transferrin receptors are maintained during each cell cycle and at each stage of nucleated erythroblast differentiation [9]. As the iron demands are met, iron uptake is decreased due to reduced transferrin receptor expression during the terminal differentiation of the cells. Ultimately, hemoglobin production ceases, as does the demand for iron. In a concerted fashion, the transferrin receptor is released from the plasma membranes of reticulocytes as one of the final steps of erythroid differentiation [10]. 

Ineffective erythropoiesis describes a group of erythroid disorders that produce fewer numbers of erythrocytes than would be expected to arise from the less mature erythroblasts present in the marrow. As a result, there exists an imbalance between the amount of iron that is endocytosed by the marrow erythroblasts and the amount of iron released into the circulation in erythrocytes [11, 12]. The concept of ineffective erythropoiesis grew from ferrokinetic studies [13]. Classic ferrokinetic studies distinguished the patterns of iron utilization during ineffective erythropoiesis from aplastic anemia, hemorrhage, or peripheral hemolysis [12]. In addition to ferrokinetics, more recent research was focused upon identifying molecular and cellular mechanisms that cause the underlying erythroid defects [14]. 

The erythroid response to tissue hypoxia is fundamental for understanding the pathophysiology of ineffective erythropoiesis. Tissue hypoxia is a common feature of anemias. Tissue hypoxia increases the expression of erythropoietin, and the erythropoietin drives the production of new erythrocytes. In cases of anemia associated with ineffective erythropoiesis, imbalance between erythrocyte supply and demand persists despite increased tissue hypoxia and increased erythropoietin. As a result, erythropoietin levels remain high, and the marrow of patients with ineffective erythropoiesis typically becomes hypercellular [15–17]. Over the course of time, the combination of tissue hypoxia, increased erythropoietin, and ineffective erythropoiesis creates a vicious cycle that may ultimately lead to a massive expansion of erythroblasts. Eventually, secondary bony pathologies [18] and extramedullary erythropoiesis [19] can also develop. Per the topic of this review, pathological iron overload in the absence of hereditary hemochromatosis is another hallmark of the disease.

Several types of erythroid defects cause significant tissue iron overload in association with ineffective erythropoiesis. The major entities are summarized in Table 1. The thalassemia syndromes (thalassemia major and intermedia) represent the most common causes of ineffective erythropoiesis. In the thalassemia syndromes, imbalances in the production of alpha- and beta-globin chains result in increased apoptosis during erythroblast maturation [14]. Iron loading, even in the absence of transfused blood, is a well-recognized complication of the disease [20]. In sideroblastic anemias, globin chain production is intact, but erythropoiesis is characterized by accumulation of iron in mitochondria that “ring” the erythroblast nucleus during maturation [21]. Intramedullary apoptosis is a feature of acquired sideroblastic anemia [22]. Some genes that regulate mitochondrial iron metabolism also cause the inherited form of sideroblastic anemia [23]. Since the inherited mitochondrial defects are not necessarily limited to hematopoietic cells, tissue iron loading in those patients may not be derived solely from ineffective erythropoiesis [24]. However, the specialization of iron, iron-sulfur, and heme trafficking in erythroblasts strongly suggests erythroid involvement in the iron loading pathology. As recently reviewed by Sheftel et al., ringed sideroblasts are associated with several erythroblast iron and mitochondrial defects [25]. It remains to be determined whether separate defects that result in sideroblastic anemia have equivalent effects upon iron homeostasis in nonerythroid tissues. A third group of disorders associated with ineffective erythropoiesis and iron overloading are called dyserythropoietic anemias [26]. The dyserythropoietic defects are distinct from others in mutations of globin and mitochondrial genes [26, 27]. Tissue iron overload is not uncommon in two of three dyserythropoietic subtypes. Pyruvate kinase deficiency results in defective glycolysis resulting in erythroblast apoptosis and peripheral blood hemolysis [28, 29]. Pyruvate kinase deficiency is being investigated as a separate cause of ineffective erythropoiesis, but iron overloading is less consistent among these patients [30]. Other erythroid disorders associated with some degree of ineffective erythropoiesis include chronic pernicious anemia, hereditary spherocytosis, and sickle cell anemia [31, 32]. However, the association between iron loading and ineffective erythropoiesis in these disorders is inconsistent [33]. 

## 3. Iron Loading and Overloading in Ineffective Erythropoiesis

Adult humans produce approximately 200 billion erythrocytes daily [34]. Each erythrocyte contains approximately 300 million molecules of hemoglobin [35]. Each hemoglobin molecule contains four heme molecules, and each heme contains a single iron moiety. Therefore, to satisfy the production of erythrocytes, 2 E20 iron molecules (20 mg) are utilized daily for erythropoiesis even in the absence of disease. The robust demand for iron is met by transferrin-bound iron in the plasma [36]. Three major sources of iron are utilized to maintain adequate levels of transferrin-bound iron: dietary iron, body iron stores, and recycled iron from senescent erythrocytes. In healthy adults, the majority of transferrin-bound iron in the plasma is generated from macrophage catabolism of mature erythrocytes in circulating blood. In steady state, the iron that is recycled from older erythrocytes is largely sufficient for the production of new erythrocytes. Phagocytosis of the older erythrocytes by macrophages results in their catabolism and the breakdown of hemoglobin. The hemoglobin-salvaged iron is loaded on transferrin for transport to marrow erythroblasts. The membrane expression of ferroportin, an iron channel protein, provides the key regulatory element of transferrin loading of iron from macrophages. Ferroportin levels on the cell membrane are regulated by another protein named hepcidin. Hepcidin acts through binding and internalization of ferroportin from the surface of iron-exporting cells [37–39]. Therefore, hepcidin is a principal regulator of the hemoglobin iron cycle. Hepcidin expression is highly regulated by a growing number of proposed mechanisms. In the steady state, broad variations in plasma levels of hepcidin are predicted among adults [40]. In iron deficient states, hepcidin expression is consistently suppressed. In ineffective erythropoiesis, hepcidin expression is less consistently suppressed [41].

**Figure 1:**
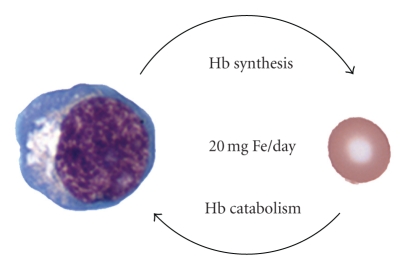
The hemoglobin iron cycle.

In a variety of conditions, the hemoglobin iron cycle (Figure 1) becomes unbalanced due to a decreased supply of iron from mature erythrocytes or an increased erythroblast demand. In most cases of anemia, the imbalance is magnified by tissue hypoxia with increased erythropoietin production. If the supply of iron from aging erythrocytes is inadequate, transferrin-bound iron must be obtained from tissue stores and the diet [42]. Increased transferrin iron loading from multiple sources is achieved when hepcidin is suppressed [43]. Erythroblast demand for iron continues until tissue demands for oxygen are satisfied, and iron stores are replenished. As shown in Table 2, several erythroid disorders are associated with imbalances between tissue hypoxia, erythropoietin, and erythropoiesis. With the exception of aplastic anemia, the erythroblast demand for iron is increased. It is presumed that hepcidin expression is reduced to meet the erythroblast demand in many, if not all of these disorders, but confirmatory studies are awaited. Despite the erythroid demand for iron, extra-erythroid loading of iron is not a typical feature of anemia or polycythemia. Remarkably, ineffective erythropoiesis is unique in causing accumulation of iron in extra-erythroid tissues to levels that are well beyond the erythroid requirements.

## 4. How Does Ineffective Erythropoiesis Cause Tissue Iron Overload?

Iron absorption is normally regulated by a combination of iron stores, inflammation, hypoxia, and erythropoietic iron demand [44, 45]. Presumably, the mechanisms that satisfy the iron appetite of immature erythroblasts are also active in ineffective erythropoiesis. Those molecular and cellular mechanisms are a focus of curiosity- and clinical-driven hematology research [46]. Along with a physiological mechanism that provides a basis for the erythroid regulator of iron, it is proposed here that pathological mechanisms may be identified that are unique to ineffective erythropoiesis. The notion of a pathological iron regulator is suggested by the unique accumulation of iron to toxic levels in patients with ineffective erythropoiesis. As discussed below, the cytokine named GDF15 could serve the role of a pathological erythroid signal. Since hepcidin plays a central role in that network, the discussion here is largely focused upon erythroid-related variables, including GDF15, which may contribute to suppressed expression of hepcidin. 

### 4.1. Iron, Iron Transport, and Iron Turnover

For many years, plasma iron parameters or the depletion of iron from the plasma compartment were actively investigated as the mechanism of signaling between erythropoiesis and iron regulation [47, 48]. Recently, it was reported that hepcidin expression correlates with transferrin saturation levels [49]. While decreased transferrin saturation could provide a mechanism for hepcidin suppression in iron deficient states, it seems less likely that the transferrin saturation levels detected in ineffective erythropoiesis (sometimes 100%) cause suppression of hepcidin. In addition to high transferrin saturation levels, there is an overall increase of holo-transferrin removal from the plasma for iron delivery to the expanded population of immature erythroblasts. Increased plasma iron turnover is increased in humans with ineffective erythropoiesis [50]. In addition, the newly incorporated iron is recycled from marrow erythroblasts rather than circulating erythrocytes. While increased plasma delivery and erythroblast recycling of iron from the erythropoietic compartment are interesting components of ineffective erythropoiesis, the significance of these features in suppression of hepcidin remains uncertain. Transferrin metabolism, saturation kinetics, plasma iron turnover, and heme recycling are all complex processes [48]. The topic remains unsettled.

### 4.2. Hypoxia and Erythropoietin in Ineffective Erythropoiesis

Tissue hypoxia directly inhibits hepcidin expression in hepatocytes. Hypoxia effects are generally independent of iron stores [46]. Since patients with severe ineffective erythropoiesis are usually anemic, tissue hypoxia may play a role in iron regulation in this disorder. Comparative data between hepatocyte cell lines and nonanemic animals exposed to hypoxic conditions consistently demonstrated a down-regulation of hepcidin production [44]. The hypoxia inducible factor/von Hippel-Lindau (HIF/vHL) pathway mediates responses to hypoxia and other cellular stressors. In normoxic, iron-sufficient conditions, an oxygen and iron-dependent prolyl hydroxylase modifies the HIF regulatory subunit named HIF1*α*. In hypoxia or iron deficiency, HIF1*α* accumulates, translocates to the nucleus, and associates with HIF1*β*, a constitutively expressed HIF subunit. The HIF heteroduplex binds promoter elements to modulate gene transcription [51]. Peyssonnaux and colleagues recently demonstrated that mice with liver-specific, conditional inactivation of HIF1*α* maintained on an iron-deficient diet develop inappropriately high levels of hepcidin [52]. Hypoxia-related changes were not specifically reported, so additional studies of this important area of research are needed. In vitro, inhibition of the prolyl hydroxylases promotes HIF1*α* stabilization, and also negatively regulates hepcidin transcription [53]. Despite the developing body of evidence in support of a direct role for hypoxia in hepcidin suppression, there is less consensus as to whether hepcidin regulation is affected through hypoxia response elements (HRE), on promoter regions of HIF/vHL pathway-dependent genes [52, 53]. Overall, it is becoming increasingly likely that HIF/vHL participates in hepcidin gene regulation networks.

A second potential mechanism for hepcidin regulation by hypoxia involves a protein named hemojuvelin (HJV). HJV is a member of the repulsive guidance molecule (RGM) family of proteins that function as coreceptors for Bone Morphogenetic Protein (BMP) signaling. The membrane form of HJV binds to type I BMP receptors and stimulates the BMPs (such as BMP2, 4 or 9) signaling. The signaling enhances the phosphorylation of SMAD signaling pathway and stimulates hepcidin transcription [54]. HJV protein is cleaved and secreted as a soluble form (sHJV) that is processed by furin-like protease (a proprotein convertase) [55–57]. sHJV acts as a repressor of BMP signaling by competing with the membrane form of HJV [58, 59]. Thus, any stimulus that leads to increased sHJV production may also reduce hepcidin expression. The generation of sHJV appears to be increased by iron deficiency and hypoxia in association with the stabilization of HIF1*α* [57, 58, 60]. Both stimuli may lead to reduced hepcidin production and increased iron absorption.

Tissue hypoxia may also regulate hepcidin and iron loading indirectly by increasing the expression of the hormone, EPO. Increased EPO is associated with erythropoietic activity, which is inversely correlated with hepcidin expression in patients with thalassemia [61]. Hepcidin was not suppressed if erythropoiesis was inhibited by EPO neutralizing antibodies, chemotherapy, or irradiation of bone marrow [62, 63]. However, high doses of EPO directly down-regulate hepcidin expression in vitro through a mechanism involving the transcription factor core element binding protein a (C/EBPa) at a cognate DNA binding site present in the hepcidin promoter [64]. While the relative contribution of direct versus indirect EPO effects upon hepcidin regulation is a matter of ongoing debate, increased expression of EPO is central to the increase in erythroid demand for iron in both effective and ineffective erythropoiesis.

## 5. Molecules Released from Erythroblasts

It is assumed in this review that erythroblasts or the process of erythropoiesis in the bone marrow of humans includes some mechanism(s) for communicating the demand for iron to distant sites in the body. For many years, scientists searched for such a mechanism without satisfaction. Along with other approaches, the avenue of genomics-based research was recently utilized for the identification of molecules released from erythroblasts that may serve as “signals” for iron regulation. Two candidate molecules (GDF15 and TWSG1) were identified by this approach. In addition to these molecules, soluble transferrin receptor has been explored as a candidate iron regulator.

### 5.1. Growth Differentiation Factor 15 (GDF15)

Thalassemic serum contains factors that suppress hepcidin expression in hepatocytes or hepatocyte cell lines [65]. Based upon the initial discovery that SMAD4 signal transduction is involved in hepcidin gene regulation [66], focus was placed upon signal transduction involving the transforming growth factor-*β* (TGF-*β*) superfamily. Using a transcriptional profiling approach during erythropoiesis, a member of that superfamily named growth differentiation factor 15 (GDF15) was discovered to be up-regulated in thalassemic serum and can suppress hepcidin expression in vitro [67]. Interestingly, GDF15 (also called, MIC-1, PLAB, PDF, PTGF-*β*, NRG-1, and NAG-1) can be regulated by p53 [68]. Since intramedullary apoptosis is frequently associated with ineffective erythropoiesis, GDF15 was explored as a lead candidate molecule as a pathological erythroid regulator of iron. In cultured human hepatocytes and hepatic cell lines, both recombinant GDF15 as well as GDF15 in the serum of thalassemia patients inhibited the expression of hepcidin. Like thalassemia syndromes, congenital dyserythropoietic anemia type I also showed high levels of serum GDF15 and inappropriate suppression of hepcidin associated with iron-overload [69]. Moreover, the high elevation of GDF15 was identified in refractory anemia with ring sideroblasts [70]. Interestingly, in vitro studies demonstrated that erythropoiesis-specific production of GDF15 was dependent upon EPO. The production of GDF15 was also stimulated by a compound that reduces mitochondrial membrane potential in erythroblasts [70]. These data support a concept that apoptotic erythroblasts produce GDF15, and that GDF15 contributes to extra-erythroid tissue iron loading due to ineffective erythropoiesis. 

It must be stressed that there is minimal evidence to date which suggests that GDF15 plays an important role in iron regulation outside the setting of ineffective erythropoiesis. Lakhal et al. demonstrated up-regulation of GDF15 in response to iron depletion using intracellular iron chelator in cell lines and a robust amount of intravenous desferrioxamine (DFO) in humans. GDF15 up-regulation occurred independently of HIF signaling, suggesting the involvement of a novel iron sensing pathway [71]. The study also reported increased levels of GDF15 in patients with iron deficiency. However, others reported conflicting results among blood donors (see [72]; unpublished data). In separate studies, Kanda et al. performed an in vivo physiological study of the relationship between GDF15, serum hepcidin, and erythropoiesis in the clinical setting of stem cell transplantation (SCT) [73]. The pre- and post-SCT serum hepcidin levels were monitored along with other factors that may affect hepcidin expression. After SCT, serum hepcidin levels showed a significant inverse correlation with markers of erythropoietic activity, such as the sTfR and the reticulocyte counts, but not GDF15 levels. Ashby et al. also performed an in vivo study for physiological erythropoiesis focusing on the relationship between plasma hepcidin, GDF15, and sTfR. Neither EPO administration nor venesection caused significant changes in GDF15 or sTfR levels (see below) despite a clear suppression of hepcidin [74]. Hence, it would surprise the authors of this review if GDF15 is determined to be the major erythroid regulator of iron in healthy adult humans. Instead, these results suggest that GDF15 contributes to hepcidin suppression and iron overloading in the pathological setting of ineffective erythropoiesis as originally proposed [67].

### 5.2. Twisted Gastrulation (TWSG1)

Erythroblast expression of a second molecule named twisted gastrulation was explored as a potential erythroid regulator of hepcidin. Expression of the TWSG1 gene was discovered as part of erythroblast transcriptome analyses. Further interest in this candidate molecule grew from the hypothesis that TWSG1 may regulate hepcidin in a similar fashion to soluble hemojuvelin (sHJV). Hemojuvelin has a key role in hepcidin regulation. Membrane HJV acts as a coreceptor for BMPs, whereas soluble HJV (sHJV) down-regulates hepcidin in a competitive way interfering with BMP signaling [57]. The TWSG1 gene product is a small, secreted cysteine-rich protein that antagonizes the interaction of bone morphogenetic proteins (BMP) with its receptor [75, 76]. Like sHJV, TWSG1 could suppress hepcidin expression by interfering with BMP signaling as a BMP antagonist. In vitro, TWSG1 protein interferes with BMP-mediated hepcidin expression in human hepatocytes. Phosphorylation studies further suggest that TWSG1 acts by inhibiting the BMP-dependent activation of SMAD-mediated signal transduction. TWSG1 secreted from erythroblasts may thus contribute to iron loading by inhibiting BMP-mediated hepcidin expression [77]. Unfortunately, assays are not yet available to measure the level of TWSG1 in human blood. Since murine hepatocytes produce BMPs and the major sites of erythropoiesis in murine thalassemia are the spleen and liver, it was proposed that this cytokine may be more active in hepcidin suppression in the murine model system [78, 79]. 

### 5.3. Soluble Transferrin Receptor (sTfR)

Approximately 80% of soluble TfR1 (sTfR) is generated during the maturation of erythroid cells [80]. Soluble TfR is a truncated form of the TfR on the surface of the cells [81]. The sTfR level indicates erythropoietic activity and iron status of the organs [82]. sTfR is increased in thalassemia [67], congenital dyserythropoietic anemia [69, 81], and sideroblastic anemia [83]. All of these observations make sTfR a strong candidate for erythroid regulation of hepcidin and iron. However, it was concluded on the basis of a transgene expression model in mice that sTfR levels neither regulate hepcidin nor iron [84].

## 6. Summary

The phrase “ineffective erythropoiesis” collectively describes a group of erythroid defects that are marked by decreased erythrocyte production despite increased early erythropoiesis. Ineffective erythropoiesis manifests a unique feature of non-transfusional iron overload in extra-erythroid tissues. This feature of secondary hemochromatosis distinguishes ineffective erythropoiesis from other causes of anemia. Further, the excess iron accumulates in parenchymal cells and negatively affects clinical outcome [85]. As such, the regulation of iron loading and overloading in ineffective erythropoiesis remains a fertile area of basic and clinical research. Physiological mechanisms that regulate iron in the context of hemoglobin production and catabolism are likely involved in both effective (normal) and ineffective erythropoiesis. In this review, we proposed that additional mechanisms or signals related to the erythroid pathology may contribute to iron overloading in other tissues. Increased expression of GDF15 from apoptotic erythroblasts is being explored in this context. Based upon the rapid pace of discovery within the field of iron biology, additional mechanisms and insights regarding the special relationships between erythropoiesis and iron regulation are predicted. 

## Figures and Tables

**Table 1:** Ineffective erythropoiesis associated with iron overload in absence of transfusion.

Erythroid disorder	Defect	Reference
Thalassemia syndromes	Globin chain imbalance.	[67, 86]
Sideroblastic Anemia (inherited or acquired)	Iron accumulation in mitochondria.	[23, 70, 87, 88]
Dyserythropoietic Anemia (Types I and II)	Nuclear and membrane defects.	[26, 27, 69]

**Table 2:** A comparison of erythroid pathologies.

Erythroid condition	Tissue iron overload	Tissue hypoxia	Increased erythropoietin	Increased erythropoiesis
*Ineffective erythropoiesis*	*Yes*	Yes	Yes	Yes
Hemolysis	No	Yes	Yes	Yes
Blood loss	No	Yes	Yes	Yes
Iron deficiency anemia	No	Yes	Yes	No
Aplastic anemia	No	Yes	Yes	No
Secondary polycythemia	No	Yes	Yes	Yes
Primary polycythemia	No	No	No	Yes
